# Nogo-B Receptor Directs Mitochondria-Associated Membranes to Regulate Vascular Smooth Muscle Cell Proliferation

**DOI:** 10.3390/ijms20092319

**Published:** 2019-05-10

**Authors:** Yi-Dong Yang, Man-Man Li, Gang Xu, Lan Feng, Er-Long Zhang, Jian Chen, De-Wei Chen, Yu-Qi Gao

**Affiliations:** 1 Institute of Medicine and Hygienic Equipment for High Altitude Region, College of High Altitude Military Medicine, Army Medical University (Third Military Medical University), Chongqing 400038, China; yydnbility@163.com (Y.-D.Y.); xg270251@163.com (G.X.); lan_eligere@163.com (L.F.); loong0810@sina.com (E.-L.Z.); jchenone@163.com (J.C.); 2 Key Laboratory of Extreme Environmental Medicine, Ministry of Education of China, Chongqing 400038, China; mmlsunshine@163.com; 3 Key Laboratory of High Altitude Medicine, PLA, Chongqing 400038, China; 4 Department of High Altitude Physiology & Biology, College of High Altitude Military Medicine, Army Medical University, Chongqing 400038, China; 5 Department of Pathophysiology, College of High Altitude Military Medicine, Army Medical University, Chongqing 400038, China

**Keywords:** VSMCs, NgBR, MAM

## Abstract

Mitochondria-associated membranes (MAM) are a well-recognized contact link between the mitochondria and endoplasmic reticulum that affects mitochondrial biology and vascular smooth muscle cells (VSMCs) proliferation via the regulation of mitochondrial Ca^2+^(Ca^2+^_m_) influx. Nogo-B receptor (NgBR) plays a vital role in proliferation, epithelial-mesenchymal transition, and chemoresistance of some tumors. Recent studies have revealed that downregulation of NgBR, which stimulates the proliferation of VSMCs, but the underlying mechanism remains unclear. Here, we investigated the role of NgBR in MAM and VSMC proliferation. We analyzed the expression of NgBR in pulmonary arteries using a rat model of hypoxic pulmonary hypertension (HPH), in which rats were subjected to normoxic recovery after hypoxia. VSMCs exposed to hypoxia and renormoxia were used to assess the alterations in NgBR expression in vitro. The effect of NgBR downregulation and overexpression on VSMC proliferation was explored. The results revealed that NgBR expression was negatively related with VSMCs proliferation. Then, MAM formation and the phosphorylation of inositol 1,4,5-trisphosphate receptor type 3 (IP_3_R3) was detected. We found that knockdown of NgBR resulted in MAM disruption and augmented the phosphorylation of IP_3_R3 through pAkt, accompanied by mitochondrial dysfunction including decreased Ca^2+^_m,_ respiration and mitochondrial superoxide, increased mitochondrial membrane potential and HIF-1α nuclear localization, which were determined by confocal microscopy and Seahorse XF-96 analyzer. By contrast, NgBR overexpression attenuated IP_3_R3 phosphorylation and HIF-1α nuclear localization under hypoxia. These results reveal that dysregulation of NgBR promotes VSMC proliferation via MAM disruption and increased IP_3_R3 phosphorylation, which contribute to the decrease of Ca^2+^_m_ and mitochondrial impairment.

## 1. Introduction

Hypoxic pulmonary hypertension (HPH) is a well-known component of pulmonary artery hypertension (PAH), which is characterized by persistent pulmonary vasoconstriction and vascular remodeling, finally leading to heart failure. Underlying the pathogenesis, excessive pulmonary vascular smooth muscle cell (VSMC) proliferation is the convergence point. Growing evidence suggests that mitochondrial dysregulation plays a vital role in VSMC proliferation [1], displaying mitochondria-induced suppression of apoptosis and activation of proliferating signaling. Interestingly, the functional and structural changes of remodeled arteries in HPH nearly completely reverse in normoxia after exposure to chronic hypoxia [2,3,4], however it is not reversed in a murine model with endothelial Bone Morphogenetic Protein Receptor Type II (BMPRII) mutations due to mitochondrial impairment [4]. Furthermore, Dichloroacetate, which can restore mitochondrial function, has proven its effectiveness in experimental models of cancer and PAH through the enhancement of glucose oxidation (GO) and the attenuation of mitochondria-induced hypoxia-inducible factor 1α (HIF-1α) signaling [5]. 

Most cancer cells and pulmonary artery smooth muscle cells (PASMCs) undergo a metabolic shift, enhancing glycolysis and suppressing GO, leading to dysregulation of mitochondria-derived reactive oxygen species (mROS), citrate, and α-ketoglutarate and induction of the proliferation-related signaling pathways, including nuclear factor of activated T cells (NFAT), voltage-gated potassium channel 1.5 (Kv1.5), and HIF-1α [5,6]. In turn, downstream factors such as HIF-1α can inhibit GO via inducing the expression of glycolysis enzymes, ultimately causing a detrimental regulatory loop to enhance proliferation. The role of GO suppression and mitochondrial abnormalities in proliferative diseases is still under active investigation. A decrease in the level of mitochondrial Ca^2+^ (Ca^2+^_m_) is one of the predominant reasons underlying this impairment. As a result, the activity of Ca^2+^-dependent GO enzymes, including pyruvate dehydrogenase, isocitrate dehydrogenase, and α-oxoglutarate dehydrogenase, is compromised, leading to GO inhibition [5]. Intriguingly, hyperproliferative VSMCs have been shown to display decreased Ca^2+^_m_ influx [7,8,9], which is regulated by the mitochondria-associated membranes (MAM). 

MAM, a synapse-like ultrastructure between the mitochondria and endoplasmic reticulum (ER), is known to transfer ER Ca^2+^ to the mitochondria through the residing inositol 1,4,5-trisphosphate receptor (IP_3_R)-glucose regulated protein 75 (GRP75)-voltage-dependent anion-selective channel 1 (VDAC1) complex. Considering that the Ca^2+^_m_ uniporter has a low Ca^2+^ affinity, Ca^2+^_m_ influx mainly relies on the proximal microdomains. In addition to Ca^2+^_m_ regulation, MAM is also involved in steroid synthesis, phospholipid metabolism, proliferation, autophagy, and mitochondrial biology [10]. Lines of constitutive proteins, kinases, and signaling pathways (such as pAkt-inositol 1,4,5-trisphosphate receptor type 3 (IP_3_R3), pAkt-phosphofurin acidic cluster sorting protein 2 [PACS2]) have been discovered at the MAM surface, which maintain close spatial proximity and biological functions [11]. Recent studies have revealed that MAM formation and functions are enhanced in several conditions, such as senescence, Alzheimer’s disease, gangliosidosis, obesity, and insulin resistance [12]. It has also been shown that enhanced MAM formation increases the apoptosis of PASMCs and ameliorates HPH [8], which it is induced by silencing Nogo-B via increase in ER Ca^2+^ flux to the mitochondria. Likewise, blocking ER stress, an upstream factor of Nogo-B, has been shown to be a potential therapeutic strategy for HPH in a murine model [9]. 

Nogo-B receptor (NgBR) resides in the ER and stimulates its ligand, Nogo-B, to regulate migration and proliferation in endothelial cells and VSMCs [13]. NgBR is required for cerebral vasculature development [14] and vascular angiogenesis [15]. It has been shown that the reduction of the NgBR level attenuates the vascular endothelial growth factor (VEGF)-induced phosphorylation of Akt, the phenotype of chemotaxis, and morphogenesis in endothelial cells, while transfection with active Akt plasmid reverses this effect. Follow-up studies suggest that the cytoplasmic domain of NgBR resembles hydrophobic pockets to bind prenylated Ras and promotes its accumulation and activation at the membrane. Recently, it was reported that the decrease of NgBR expression promoted PASMC proliferation through regulation of reactive oxygen species (ROS) production in an intrauterine pulmonary hypertension (IPH) model [16]. However, the underlying mechanism remains unclear. 

This study aims to explore the role of NgBR in MAM and subsequent mitochondrial function in VSMCs. Here, we analyzed the alteration in NgBR expression in vivo and in vitro under hypoxia-renormoxia and elucidated its function in MAM formation and MAM-associated pAkt signaling. Our results provide a better understanding of the role of NgBR in mitochondrial dysfunction-related diseases.

## 2. Results

### 2.1. NgBR Expression Was Downregulated in the Thickened Pulmonary Arteries of HPH Rat Model and Hypoxic VSMCs In Vitro

**Figure 1:**
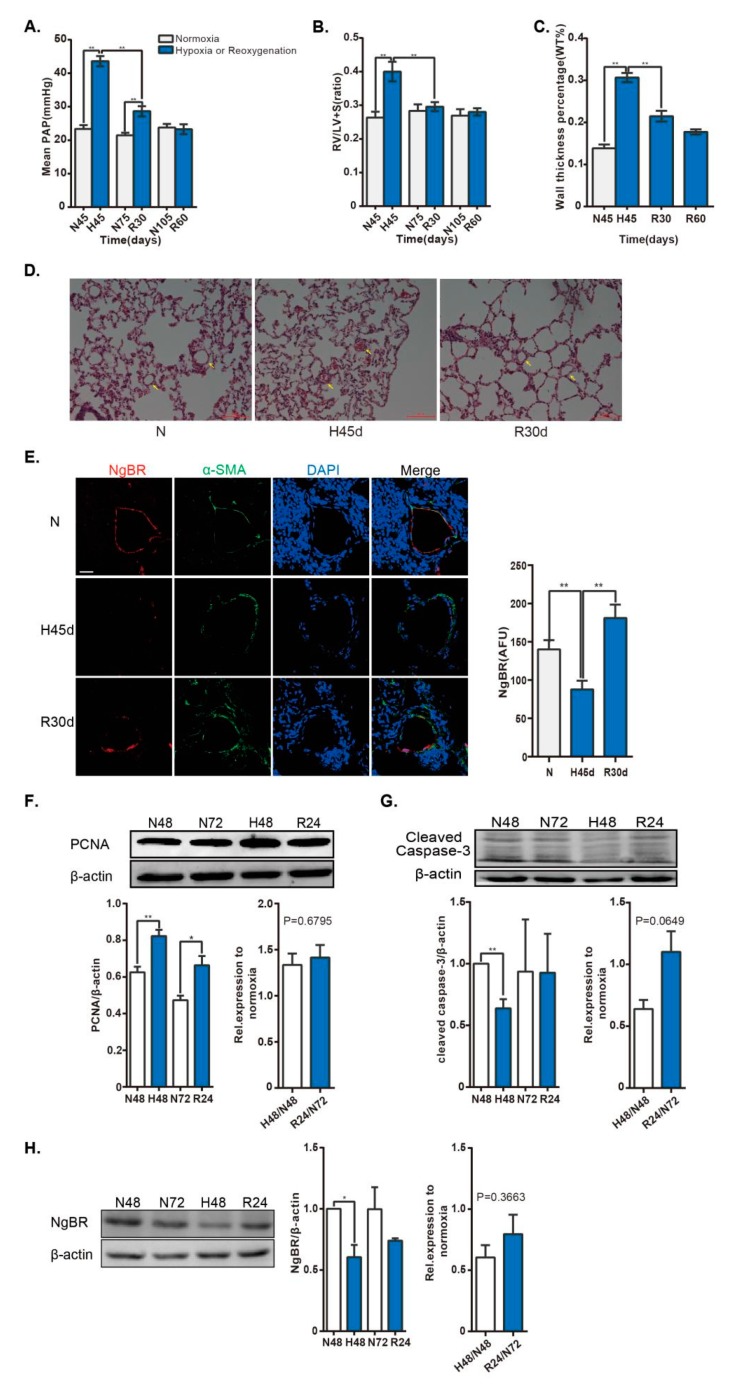
NgBR expression is downregulated in the pulmonary artery of HPH rat model and in vascular smooth muscle cells (VSMCs) exposed to hypoxia. Rats were housed in hypobaric hypoxia (10.0% O_2_) for 45 days, and then exposed to reoxygenation at different times. Pulmonary vascular remodeling was evaluated by measuring the (**A**) mPAP and (**B**) weight ratio of RV/ (LV + S). (**C**,**D**) Histological images of distal pulmonary arteries (arrows)stained with hematoxylin and eosin. Scale bar = 100 µm. The percent medial wall thickness was analyzed using an Olympus IX-70 microscope. n = 44–50 pulmonary arteries (35–100 µm) from three animals/group. (**E**) The small pulmonary arteries (30–140 µm) were co-stained with α-smooth muscle actin (α-SMA; green) and NgBR (red). Nuclei were stained with DAPI (blue). Results were calculated as a relative arbitrary immunofluorescence unit (AFU) using FV10-ASW3.1 software. n = 38–54 pulmonary arteries from three animals/group. Scale bar = 30 µm. The VSMC cell line, A10, was exposed to 4% O_2_ for 48 h, followed by exposure to 21% O_2_ for 24 h. (**F**,**G**) Cell proliferation was evaluated by determining PCNA expression. n = 4. Apoptosis was detected by analyzing cleaved-capase-3 expression. n = 3. ** *p* < 0.01 H48 versus N48, R24 versus N72. (**H**) NgBR expression was normalized to β-actin. n = 3. * *p* < 0.05.

HPH is a well-known proliferative VSMC model. A spontaneous regression of hypoxia-induced pulmonary vascular remodeling has been observed under normoxia after hypoxia exposure [3,4]. Here, we showed that the mean pulmonary arterial pressure (mPAP) and right ventricular hypertrophy index were elevated during hypoxia and gradually reduced following reoxygenation (Figure 1A,B). Concomitantly, the wall thickness percentage, which increased during hypoxia, was decreased after reoxygenation (Figure 1C,D). Taken together, we established an HPH model and a subsequent renormoxia-induced spontaneous regression model, as previously described. In contrast, immunofluorescence staining results revealed that NgBR expression was reduced in hypoxia and enhanced after reoxygenation in the pulmonary arteries (Figure 1E). Next, a proliferating VSMC model was observed in hypoxia exposure in vitro. The proliferating cell nuclear antigen (PCNA) level was elevated in hypoxia. Correspondingly, the expression of cleaved-caspase-3, a critical executioner of apoptosis, was attenuated under hypoxia. However, the PCNA level still increased in reoxygenation compared to the corresponding control, and the ratio of PCNA level in reoxygenation to corresponding time point in normoxia was not reduced compared with that in hypoxia group, despite of the tendency for elevation in the ratio of cleaved-caspase-3 level (Figure 1F,G). Additionally, the protein level of NgBR was reduced under hypoxia, but not recovered in reoxygenation, coincided with the unchanged PCNA level (Figure 1H). Altogether, downregulation of NgBR was observed in the proliferating VSMCs in vivo and in vitro. 

### 2.2. Downregulation of NgBR Recapitulates the Hypoxia-Induced Proliferation of VSMCs in Normoxia

**Figure 2:**
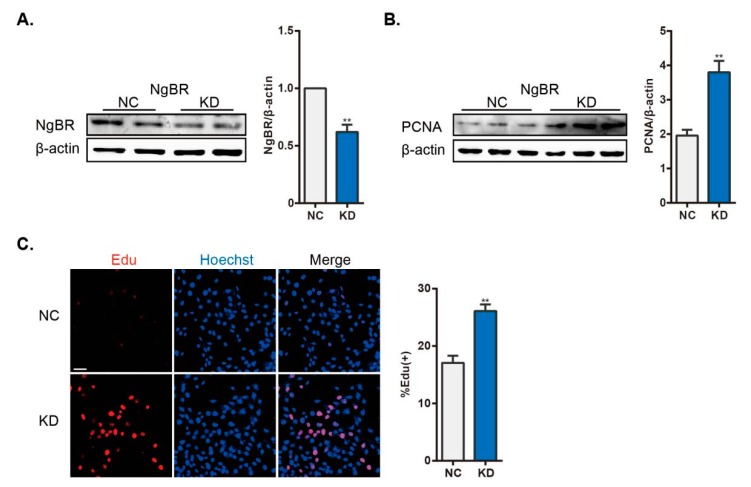
Increased proliferation in NgBR KD cells under normoxia. (**A**) The efficiency of shNgBR transfection was verified at 48 h post transfection, the statistical comparisons histogram from three independent experiments. (**B**) Cell proliferation was assessed by evaluating PCNA expression, n = 3. (**C**) Proliferating cells were stained with EdU (red) and nuclei were labeled with Hoechst (blue). n = 33 pictures/group. Scale bar = 40 μm. Percentage of EdU-positive nuclei was calculated using FV10-ASW3.1 software. ** *p* < 0.01.

As mentioned above, we established a model that mimics hypoxia-induced proliferation of VSMCs. NgBR expression was then suppressed by shRNA transfection of VSMCs. shNgBR cells exhibited nearly 50% reduction of NgBR expression compared with control cells (Figure 2A). In keeping with previous work [16], hyperproliferation was enhanced in NgBR knockdown (KD) cells, which showed increased percentage of EdU-positive cells and increased PCNA expression (Figure 2B,C). In summary, downregulation of NgBR expression mimicked hypoxia-induced proliferation under normoxia.

### 2.3. Downregulation of NgBR Disrupts MAM Formation

**Figure 3:**
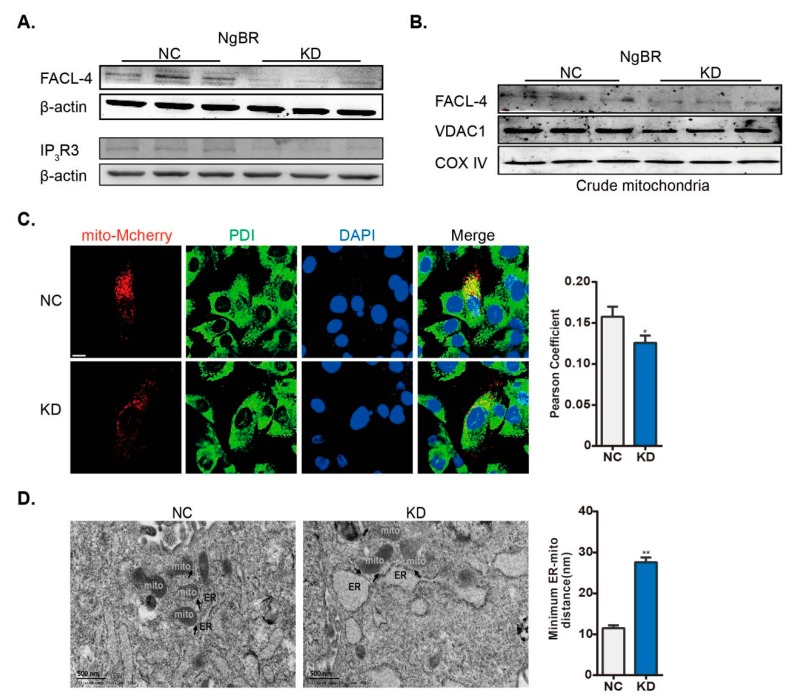
NgBR KD cells show disrupted ER-mitochondria interaction. shControl- and shNgBR-transfected cells were harvested at 48 h post transfection. MAM formation was analyzed by evaluating the expression of MAM-related proteins in (**A**) total cell lysates (FACL-4 and IP_3_R3) and (**B**) crude mitochondrial extracts (FACL-4 and VDAC1); β-actin and COX IV were utilized as the loading controls, respectively. n = 3. (**C**) Cells were incubated with plasmid expressing mCherry fused with a targeting sequence of subunit IV of COX prior to shRNA transfection. PDI was used as an ER marker. Nuclei were stained with Hoechst (blue). Pearson’s correlation coefficient was calculated to measure colocalization of ER and mitochondria on mCherry positive cells. n > 60 cells/group. Scale bar = 10 μm. (**D**) Transmission electron microscopy of negative control (NC; left) and NgBR knockdown KD (right) VSMCs. Arrowheads indicate the MAMs. The minimum ER-mitochondrial distance was quantified. n > 80 ER-mitochondria contact points/group. Scale bar = 500 nm. * *p* < 0.05, ** *p* < 0.01.

It has been reported that the disruption of MAM represses mitochondrial function, which contributes to PASMC proliferation [8]. As mentioned above, downregulation of NgBR stimulated VSMC proliferation. Accordingly, we next evaluated the potential effect of NgBR on MAM formation. We found that the protein level of IP_3_R3 and Fatty-Acid-Coenzyme A Ligase, Long-Chain 4 (FACL-4) was reduced in total lysates, and the levels of FACL-4 and VDAC1 were decreased in the crude mitochondrial fractions from NgBR KD cells (Figure 3A,B). Next, the co-immunostaining of Protein Disulfide Isomerase (PDI) (ER marker) and mitochondria-targeted mCherry was performed to evaluate the formation of MAM, and the Pearson’s correlation coefficient (ER-mitochondria interaction) was reduced in NgBR KD cells (Figure 3C). In the end, we also visualized MAM formation through the electron microscope images, and a lower degree of ER apposition to the mitochondria was found in NgBR KD cells (Figure 3D). Taken together, downregulation of NgBR disrupted mitochondria-ER interaction under normoxia.

### 2.4. Downregulation of NgBR Promotes pAkt-IP_3_R3 Signaling at the MAM Surface and Inhibits Ca^2+^_m_ Level

**Figure 4:**
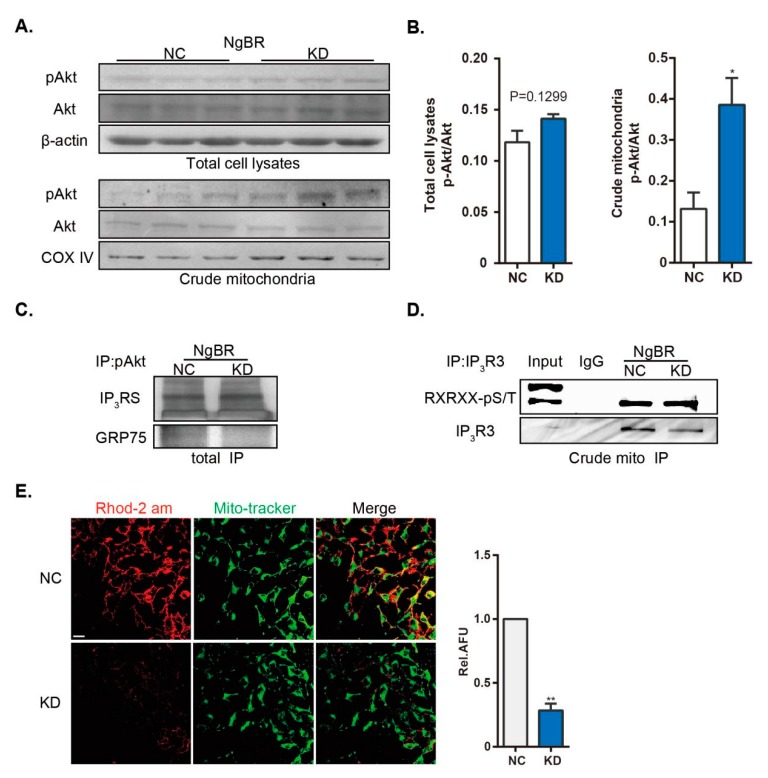
Downregulation of NgBR enhances pAkt signaling at the MAM surface and reduces Ca^2+^_m_. The phosphorylation of Akt (Ser473) and total Akt level were detected in (**A**,**B**) total cell lysates and crude mitochondrial extracts. n = 3. (**C**) IP of pAkt was performed from a total cell homogenate, and MAM-localized proteins (IP_3_Rs and GRP75) were detected. (**D**) Phosphorylation of endogenous IP_3_R3 (Akt substrate) was detected in the immunoprecipitates of IP_3_R3 from crude mitochondrial extracts. (**E**) Representative confocal microscopy images showing co-staining of Rhod-2 AM (red) and MitoTracker Green (green). Results were calculated as relative AFU using ImageJ. n > 20 pictures/group from three independent experiments. Scale bar = 20 μm. * *p* < 0.05, ** *p* < 0.01.

To test the hypothesis that NgBR affects pAkt-IP_3_R3 signaling at the MAM surface, we determined the alterations of pAkt/IP_3_R3 levels in crude mitochondrial extracts and whole homogenates. In accordance with previously published results [16], the ratio of pAkt to total Akt level was not altered in NgBR KD cells (Figure 4A,B). However, downregulation of NgBR increased the level of pAkt in crude mitochondrial fractions (Figure 4A,B), which indicates the alterations in MAM [11]. Next, we detected more pAkt-IP_3_Rs complex formation in the immunoprecipitates from NgBR KD cells, despite a decrease in the level of immunoprecipitated GRP75 (Figure 4C). GRP75 resembles a “bridge” linking IP_3_Rs and VDAC1 proteins, which are separately anchored at the ER and mitochondrial membrane. In other words, shNgBR cells displayed less communication between the ER and mitochondria as mentioned above, but more interaction of pAkt with MAM residing IP3Rs. Phosphorylation of IP3R3, a substrate of pAkt at the MAM, specially mediates the transfer of Ca^2+^ from the ER to the mitochondria through MAM, thereby inhibiting cell death and promoting cell survival [17,18,19]. We confirmed the enhanced phosphorylation of IP_3_R3 in the crude mitochondrial extracts from NgBR KD cells, although IP_3_R3 expression was reduced (Figure 4D). Finally, we analyzed the level of Ca^2+^_m_, a well-known indicator of MAM. Ca^2+^_m_ level was assessed by staining with Rhod-2 AM, a mitochondria-specific Ca^2+^ fluorophore, as shown by its colocalization with the mitochondrial marker MitoTracker Green. The results showed that the level of Ca^2+^_m_ was attenuated in NgBR KD cells (Figure 4E).

### 2.5. Downregulation of NgBR Impairs MAM-Regulated Mitochondrial Function

**Figure 5:**
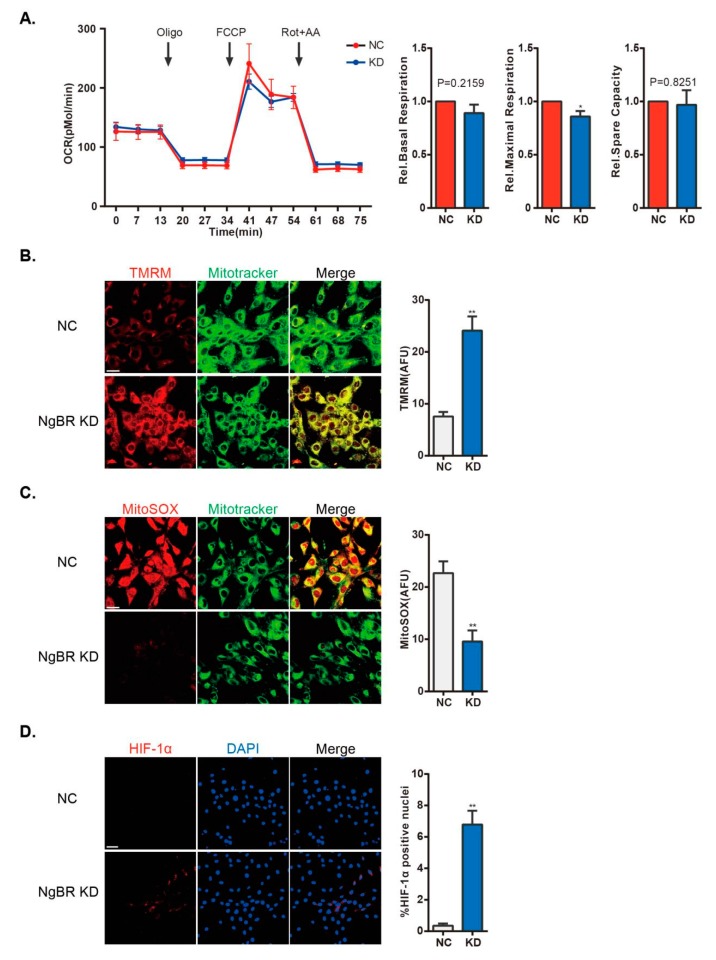
Downregulation of NgBR compromises mitochondrial function in normoxic VSMCs. (**A**) Representative line and bar graphs of OCR of NS and NgBR KD cells. n = 20–21 wells from four individual experiments. mROS generation and ΔΨ were assessed by (**B**) TMRM fluorescent probes (n = 13–14 pictures/group) and (**C**) MitoSOX™ Red (n = 13–16 pictures/group). Results were calculated as AFU using ImageJ. Scale bar = 20 µm. (**D**) Representative confocal microscopy images showing staining of HIF-1α (red) and DAPI (nuclear stain; blue). Percentage of HIF-1α-positive nuclei was calculated using FV10-ASW3.1 software. n > 30 pictures/group. Scale bar = 40 µm. * *p* < 0.05, ** *p* < 0.01.

Ca^2+^_m_ is required for calcium-sensitive GO enzymatic activity [8]. Accumulation of Ca^2+^_m_ alters mROS generation and ΔΨ, and elicits integrated mitochondrial signaling, such as activation of NFAT and HIF-1α in hyperproliferative VSMCs. Decrease of Ca^2+^_m_ impairs mitochondrial oxidative respiration. As shown, NgBR KD cells showed impaired maximal respiration (Figure 5A). Mitochondrial hyperpolarization and decline of mROS generation play key roles in mitochondrial remodeling in proliferating, anti-apoptotic PAH PASMCs [6,7,9]. We detected an increase in the ΔΨ value in NgBR KD cells (Figure 5B). We also found that mROS generation was decreased in NgBR KD cells using MitoSOX^TM^ Red, a mitochondrial superoxide indicator (Figure 5C). Impaired mitochondria activate downstream signaling pathways such as HIF-1α and NFATc2, and downregulates the expression of Kv1.5 in cancer cells and proliferating VSMCs. Translocation of HIF-1α to the nucleus was detected by immunofluorescence staining in NgBR KD cells. As shown in Figure 5D, HIF-1α was activated in shNgBR cells. HIF-1α activation has been shown to promote glycolysis and suppress mitochondrial biosynthesis [20]. Thus, it creates a feed-forward/feedback loop to impair mitochondrial function, resulting in prolonged cell proliferation.

### 2.6. NgBR Overexpression Inhibits Hypoxia-Induced Proliferation of VSMCs via Restoring Mitochondria-ER Interaction and Attenuating pAkt Signaling

**Figure 6:**
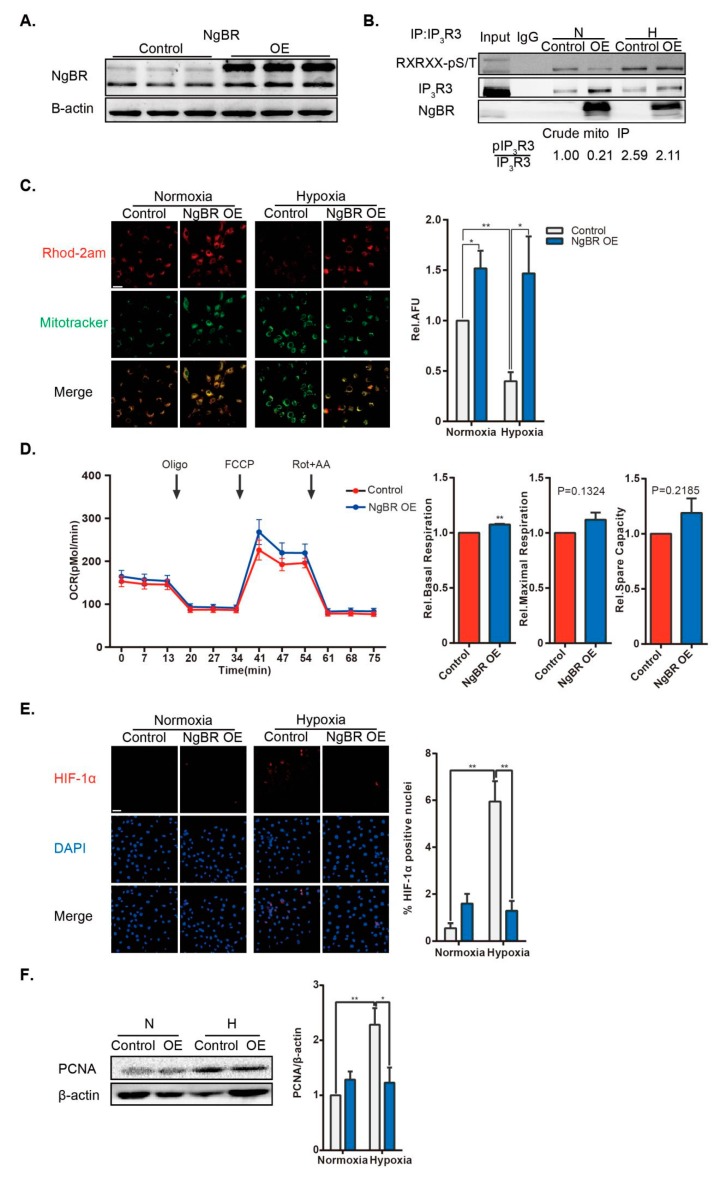
NgBR overexpression (OE) suppresses cell proliferation through enhancing mitochondria-ER communication and reducing phosphorylation of IP_3_R3. (**A**) The transfection efficiency of NgBR OE plasmid was verified at 48 h post transfection. n = 3 (**B**) Representative images from three experiments show phosphorylation of endogenous IP_3_R3 (Akt substrate) in the immunoprecipitates of IP_3_R3 from crude mitochondrial extracts. (**C**) Representative confocal microscopy images show co-staining of Rhod-2 AM (red) and MitoTracker Green (green). Results were calculated as relative AFU using ImageJ. n = 20 pictures/group from four independent experiments. Scale bar = 20 µm. (**D**) Representative line and bar graphs of OCR of control and NgBR OE cells under normoxia. n = 15–16 wells from three individual experiments. (**E**) Representative confocal microscopy images showing staining of HIF-1α (red) and DAPI (nuclear stain; blue). Percentage of HIF-1α-positive nuclei was calculated using FV10-ASW3.1 software. n > 25 pictures/group from three separate experiments. Scale bar = 40 µm. (**F**) Cell proliferation was assessed by evaluating PCNA expression, n = 4. * *p* < 0.05, ** *p* < 0.01.

To confirm the role of NgBR in MAM and its function in VSMC proliferation, we performed a rescue experiment of NgBR overexpression (OE) in hypoxic VSMCs. The transfection efficiency of NgBR OE plasmid was verified in VSMCs (Figure 6A). Although the expression of the MAM-related protein IP_3_R3 was upregulated in NgBR OE group, phosphorylation of IP_3_R3 was diminished in crude mitochondrial immunoprecipitates from hypoxic NgBR OE cells compared with that in the hypoxic control group (Figure 6B). As expected, NgBR OE abrogated the hypoxia-induced Ca^2+^_m_ suppression (Figure 6C) via enhancing mitochondria-ER communications and inhibiting localized phosphorylation of IP_3_R3. Basal respiration was enhanced in NgBR OE groups (Figure 6D). Taken together, NgBR OE resulted in Ca^2+^_m_ elevation and inhibition of downstream HIF-1α activation in hypoxia (Figure 6E), thereby suppressing the hypoxic proliferation of VSMCs (Figure 6F). 

**Figure 7:**
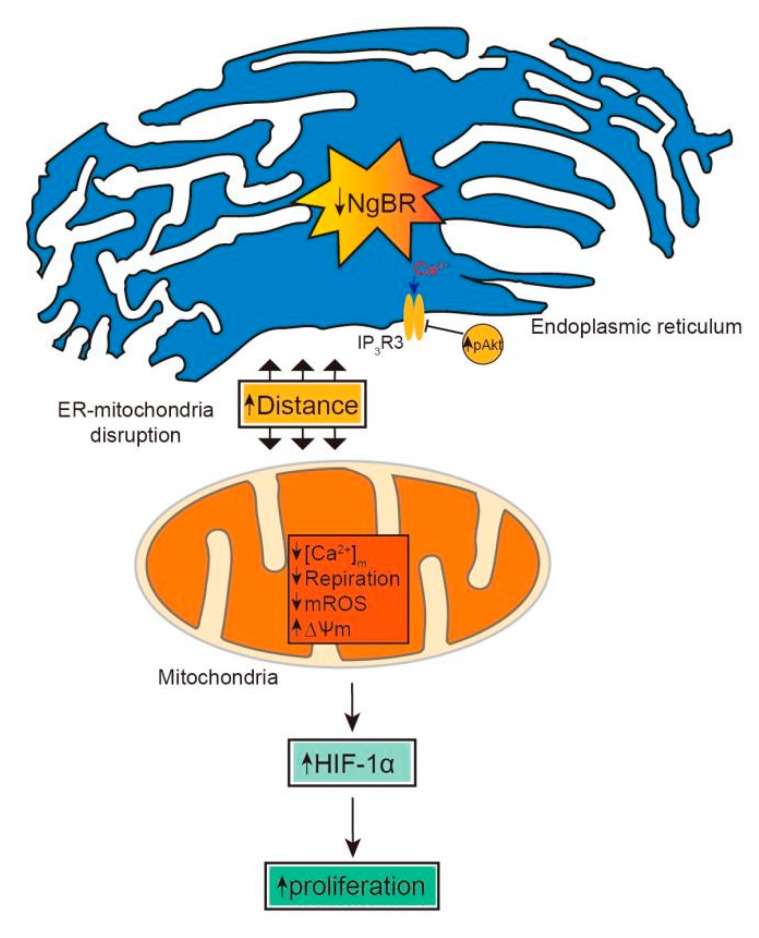
A schematic diagram showing how NgBR is involved in MAM-regulated VSMC proliferation. Downregulation of NgBR disrupts the mitochondria-ER unit and increases MAM-associated pAkt-IP_3_R3 signaling, resulting in the suppression of mitochondrial function. Consequently, mitochondria-induced HIF-1α signaling is activated to stimulate cell proliferation.

Collectively, these data provide a functional link between NgBR, MAM-mediated mitochondrial impairment, and the pathogenesis of VSMC proliferation (Figure 7).

## 3. Discussion

Mitochondrial dysfunction is a well-known contributor of VSMC proliferation and vascular remodeling, but the mechanism by which it occurs remains unclear. In this study, we demonstrated that NgBR expression is suppressed in vivo and in vitro under hypoxia. Downregulation of NgBR promotes the proliferation of hypoxic VSMCs through mitochondrial impairment, which is caused by a decreased level of Ca^2+^_m_. Disruption of MAM and enhanced pAkt/IP_3_R3 signaling lead to attenuation of mitochondrial Ca^2+^ influx. These results indicate that NgBR plays an important role in mitochondrial impairment from excessive VSMC proliferation.

PAH is a fatal disease characterized by persistent vascular contraction, excessive proliferation of VSMCs, and aberrant secretion of endothelial cells. Using a specific spontaneous regression model of HPH, we investigated the role of NgBR in VSMC proliferation. Contrary to the increased level of Nogo-B in HPH, we observed that exposure to hypoxia suppressed NgBR level in the remodeled pulmonary arteries, while NgBR expression was increased after normoxic recovery. The pathogenesis of “recovery” has received particular interest. Not only is the proinflammatory and pro-proliferative environment reversed during the recovery [21], but there is also a change in the characteristic phenotype of VSMCs towards a proapoptotic phenotype [22]. This observation enhances our understanding on the relationship between NgBR and VSMC proliferation. In our in vitro model, VSMCs proliferation was not reversed in reoxygenation. This may be explained by the time of renormoxia exposure not being sufficient to reflect the restoration of excessive proliferation, as our observations suggested that wall thickness in remodeled arteries was nearly reversed to that in normoxia until two-months recovery later. Here, we discovered that exposure to hypoxia suppressed NgBR expression, and downregulation of NgBR mimicked hypoxia-induced proliferation in vitro. NgBR OE attenuated this effect. 

ROS play a critical role in VSMC proliferation and endothelial dysfunction, and negatively regulates NgBR expression. Elevation of ROS has been shown to inhibit NgBR expression in normal PASMCs exhibiting hyperproliferation, while scavenging ROS restored the reduced NgBR expression in PASMCs in a model of IPH [16]. This negative feedback regulation also pertains to IPH-vascular endothelial cells. The similarity in compromised NgBR expression between IPH and HPH suggests possible common upstream pathways, given that hypoxic PASMCs show sustained increase of ROS production. Other PH models may display downregulated NgBR expression. 

We next investigated the role of NgBR in mitochondrial function. Mitochondrial hyperpolarization has been well described in the proliferating PASMCs of PH patients and animal models [5]. Likewise, NgBR KD VSMCs show mitochondrial hyperpolarization with reduced Ca^2+^_m_ influx. mROS generation is a byproduct of unpaired electron transfer in complex I and complex III during oxidative phosphorylation. Despite controversial conclusions regarding the elevation or attenuation of mROS production in PH-PASMCs, it is clear that mROS plays a principal role in the development of mitochondrial abnormalities through affecting redox-sensitive proteins, such as HIF-1α and Kv 1.5. Our data revealed that mROS production was decreased in NgBR KD VSMCs. We also detected a decline of Ca^2+^_m_ in NgBR KD cells. Attenuation of Ca^2+^_m_ influx has a causal role in decreased dehydrogenases activities in the Krebs cycle, thereby inhibiting mitochondrial respiration. NgBR OE enhanced mitochondrial respiration through an increase in Ca^2+^_m_. Unexpectedly, basal respiration was not reduced while NgBR was downregulated. Basal respiration includes two consumptions: mitochondrial oxidative phosphorylation and proton leak. Recent work suggests that downregulation of NgBR contributes to mitochondrial dysfunction due to a breakdown of the glycosylation pathway on part of oxidative phosphorylation system subunits [23]. This may lead to a perturbation of the electron transference in respiratory chain, and subsequently abnormalities of proton leak. Thus, that may cover up the reduction of mitochondrial oxidative phosphorylation. The above results suggest that downregulation of NgBR disturbs mitochondrial function.

Next, we found that downregulation of NgBR led to the activation of downstream HIF-1α under normoxia. Integration of abnormalities in the mitochondria activates downstream effectors, such as HIF-1α, which is induced at both transcriptional and protein levels in PASMCs and pulmonary arterial endothelial cells obtained from patients with pulmonary hypertension and animal models. Heterozygous HIF-1α^+/–^ mice subjected to chronic hypoxic exposure remarkably lack elevated pulmonary arterial pressure and right ventricular hypertrophy [24]. Reduced mROS relieves the inhibition of HIF-prolyl-hydroxylases and alleviates p53 activity, which inhibits HIF-1 activation at both transcription and protein levels. Concomitantly, HIF-1α itself inhibits mitochondrial biology via decreasing mitochondrial biogenesis and inducing the expression of glycolytic enzymes, in the same way that pyruvate dehydrogenase (PDH) kinase phosphorylates and inhibits PDH, ultimately causing a detrimental regulatory loop. 

We then explored a causal factor for mitochondrial impairment and showed that NgBR mediates communications between the ER and mitochondria. Recently, ER stress and mitochondrial dysfunction have been implicated in the progress of various cardiovascular diseases, including cardiac hypertrophy, ischemic heart disease, pulmonary hypertension, and atherosclerosis [5,25]. Several intriguing studies link these two fundamental cellular processes and provide novel strategies on restoring mitochondrial function [9,12]. MAM, a specialized microdomain of the ER membrane in proximity to the mitochondria, specially conducts ER Ca^2+^ transmission to the mitochondria. Grp75 is required for this ultrastructure integrity, and induces mitochondrial dysfunction under pathological status [26,27]. We found that the interaction between Grp75 and pAkt was downregulated in NgBR knock-down cells, this indicates a reduction of MAM. Our data here link the two constitutive cellular events via NgBR, and show that downregulation of NgBR reduces ER-mitochondria interaction under normoxia, causing reduced Ca^2+^_m_.

Apart from the mechanistic regulation of ER-mitochondria distance, we reported a potential regulatory role of NgBR in pAkt signaling at the MAM surface. Akt, a pivotal regulator of localized proteins at the MAM surface, targets several substrates, such as IP_3_Rs, hexokinase 2, and PACS2, followed by regulation of mitochondrial biology [11]. Intriguingly, we found that downregulation of NgBR promoted pAkt/IP_3_R3 signaling in MAM. Depletion of NgBR inhibits VEGF-stimulated phosphorylation of Akt in human umbilical vein endothelial cells, contributing to diminished cell migration and morphogenesis. A reverse effect by constitutive Akt activation demonstrates that Akt is a downstream target of NgBR. However, we found no discernible changes in pAkt/Akt level following NgBR downregulation in VSMCs, which is in agreement with a recent report [16]. Surprisingly, downregulated NgBR expression enhanced phosphorylation of Akt in the crude mitochondrial extracts, thereby increasing the level of phosphorylated IP_3_R3 and further reducing the transfer of ER Ca^2+^ into the mitochondria. The mechanism of NgBR-induced subcellular Akt activation remains unclear. Recently, NgBR was reported to recruit non-caveolae-rich lipid raft subdomains associated with H-Ras localization at the cell membrane [28]. Accumulation of H-Ras at the membrane is a canonical upstream activator of the phosphatidylinositol-4,5-bisphosphate 3-kinase-Akt pathway leading to tumorigenesis and tumor resistance. In addition to plasma membrane localization, Ras proteins also activate Akt in other cellular compartments, such as the ER and Golgi [29,30]. As expected, several membrane Ras family proteins have been recognized in isolates from both the brain and liver MAM by depth proteomic analyses [31]. Therefore, it may explain the effect of subcellular pAkt activation. However, the mechanism by which NgBR downregulation activates MAM-associated pAkt signaling, by multiple steps or direct binding, needs to be explored in future studies. 

There are certain limitations in our study. First, we used the VSMC cell line A10 instead of primary PASMCs for studying the role of NgBR in MAM and VSMC proliferation. However, the amount of subcellular fraction that can be obtained from primary PASMCs is limited. Hence, we utilized the vascular smooth muscle-derived cell line to acquire enough subcellular extracts, and established a hypoxia-induced proliferative model that displayed downregulated NgBR expression, in keeping with the results of HPH. Furthermore, MAM has a well-defined role in related proliferative/apoptotic conditions, such as PH-PASMCs, cancer, and hypoxia-reoxygenation injury [32,33]. In the present study, we demonstrate that downregulation of NgBR triggers pseudohypoxic disruption of MAM.

In summary, we report a novel role of downregulated NgBR expression in mitochondrial impairment and VSMC proliferation through suppressing MAM formation and promoting MAM-associated pAkt signaling. A better understanding about the mechanisms of NgBR is required, which may have potential implications in the treatment of vascular remodeling and mitochondrial dysfunction-related diseases.

## 4. Materials and Methods

### 4.1. Chemicals

Anti-pAkt1/2/3 (Ser473; sc-7985), IP_3_R3 (sc-7277), and NgBR (sc-138044) were purchased from Santa Cruz Biotechnology (Dallas, TX, USA). Antibodies against proliferating cell nuclear antigen (PCNA; BM0104), VDAC1 (PB0478), GRP75 (PB0668), and IP_3_R1 (PB0223) were obtained from Boster (Pleasanton, CA, USA). Antibodies against fatty acid-CoA ligase 4 (FACL-4; ab155282), cytochrome c oxidase complex IV (COX IV; ab 14744), and NgBR (ab168351) were acquired from Abcam (Cambridge, Cambs, UK). Antibodies against IP_3_Rs (#3763), phospho-(Ser/Thr) Akt substrate (#9611), and cleaved caspase-3 (#9664) were purchased from Cell Signaling Technology (Danvers, MA, USA). Anti-Akt (60203-2-Ig) was purchased from proteintech (Wuhan, Hubei, China). Anti-IP_3_R3 (610312) was obtained from BD Biosciences (Franklin Lakes, NJ, USA). Anti-HIF-1α (NB100-134) was obtained from Novus Biologicals (Littleton, CO, USA). Anti-β-actin (bsm-33036M) was obtained from Bioss (Woburn, MA, USA). Horseradish peroxidase (HRP)-labeled goat anti-rabbit/mouse IgG (H+L) was purchased from Boster. Rhod-2 AM (R1244), tetramethylrhodamine methyl ester (TMRM) reagent (I34361), and MitoSOX™ Red (M36008) were obtained from Thermo Fisher Scientific (Waltham, MA, USA). Protein A/G magnetic beads were purchased from Bio-Rad Laboratories (Hercules, CA, USA). 5-Ethynyl-2′-deoxyuridine (EdU) detection kits (C10310-3) were purchased from RiboBio (Guangzhou, Guangdong, China). Mitochondrial separation reagent (C3601) was obtained from Beyotime (Beijing, China). 

### 4.2. Animals

Animal experiments were approved by the Laboratory Animal Welfare and Ethics Committee of the Army Medical University (Chongqing, SCXK 2012-0011, 01082012, China). Adult male rats (180–200 g) were randomly divided into 6 groups (H45d, R30d, R60d, N45d, N75d, and N105d) and fed under normoxia (21% O_2_) or hypobaric hypoxia (10.0% O_2_) for the indicated days. Prior to lung isolation, the mean pulmonary artery pressure (mPAP) was measured as previously described [34]. The right ventricle (RV) free wall and the left ventricle plus septum (LV + S) were separated, and the weight ratio of RV/(LV + S) was calculated as an index of RV hypertrophy. Rats were anesthetized with urethane and perfused with 4% paraformaldehyde (PFA) in phosphate-buffered saline (PBS). The lung tissues were fixed with 4% PFA overnight at 4 °C and then embedded in optimal cutting temperature (OCT) compound. Following this, 5-μm-thick sections were made for hematoxylin and eosin staining. Images were captured using an Olympus IX-70 inverted microscope (Olympus, Tokyo, Japan). The percent medial wall thickness was analyzed as previously described [34]. Distal pulmonary arteries (50–300 μm) were identified and measured at the two ends of the shortest external diameter of the distal pulmonary arteries, and the average was taken ([2 × wall thickness/external diameter] × 100).

### 4.3. Cell Culture

The VSMC cell line A10 was purchased from the American Type Culture Collection and cultured in high-glucose Dulbecco’s modified Eagle’s medium supplemented with 10% fetal bovine serum. Experimental groups (H48 and R24) were exposed to hypoxia (4% O_2_) for 48 h, followed by exposure to normoxia (21% O_2_) for 24 h. Control groups were maintained under normoxia (21% O_2_) for 48 and 72 h. Hypoxia was established by using a mixture of ultra-high purity gases (5% CO_2_, 4% O_2_, and 91% N2) in a 37 °C hypoxia workstation (InvivO2; Baker Ruskinn, Sanford, ME, USA). Most experiments involving hypoxic groups, including Rhod-2 AM staining and cells collection for crude mitochondrial extracts were performed in the hypoxia workstation.

### 4.4. Transient Transfection with Plasmids

Short hairpin RNA (shRNA) plasmids targeting NgBR were designed with the following sequences: (sense) 5′-CGGCAGTATGCAGCTTGTGAA-3′, (antisense) 5′-TTCACAAGCTGCATACTGCCG-3′, which was provided by GeneChem (Shanghai, China), as well as an NgBR overexpression (OE) plasmid. A non-target plasmid was generated as a control. Plasmid transfection was performed using Lipofectamine 3000 (Thermo Fisher Scientific) according to the manufacturer’s instructions. 

### 4.5. Subcellular Fraction

Cells (1–2 × 10^7^) were harvested, trypsinized, washed once with cold PBS, and centrifuged at 235× *g* for 10 min. Cell pellets were resuspended in mitochondrial separation reagent (Beyotime) on ice for 15 min. The suspension was homogenized with a total of 16 strokes using a pre-cooled glass homogenizer and centrifuged at 750× *g* for 10 min. The pellet contained nuclei and intact cells. The acquired supernatant was further centrifuged at 11,000× *g* for 10 min. All centrifugation procedures were performed at 4°C. Supernatants (crude mitochondrial fraction) were collected [35].

### 4.6. Immunoprecipitation

Total cell lysates were obtained by re-suspending cell pellets in immunoprecipitation (IP) buffer (20 mM Tris (pH 7.5), 150 mM NaCl, and 1% Triton X-100) supplemented with a protease inhibitor tablet (Roche, Mannheim, Germany) and phosphatase inhibitor (Boster). Crude mitochondrial extracts were obtained as mentioned above. Specialized antibodies, including pAkt1/2/3 (sc-7985) and IP_3_R3 (C-20) antibodies (Santa Cruz Biotechnology) and control antibodies, including goat and rabbit IgG (Beyotime), were incubated with protein A/G magnetic beads at room temperature for 15 min, prior to incubation with lysates overnight at 4 °C. Then, 1× sodium dodecyl sulfate buffer was added and the precipitated immune complex was boiled at 65 °C for 25 min, following a previously described protocol [11]. 

### 4.7. Western Blotting

Equal amounts of protein samples were separated on 10% sodium dodecyl sulfate-polyacrylamide gels and transferred to polyvinylidene difluoride membranes (Bio-Rad Laboratories). After blocking with skimmed milk powder at room temperature for 1 h, primary antibodies were added and incubated overnight at 4 °C. The bands were washed with Tris-buffered saline with Tween 20 and incubated with HRP-labeled secondary antibodies at room temperature for 1 h. The immunoreactive bands were visualized using NBT/BCIP (Thermo Fisher Scientific). β-Actin and COX IV were used as the loading controls for total lysates and crude mitochondrial extracts.

### 4.8. Electron Microscopy

Detailed assessment of the morphology of mitochondria and ER was performed using a scanning transmission electron microscope (Libra 200 FEG, Zeiss, Oberkochen, Germany; 200 kV). Morphological calculations were performed according to a previously established method using ImageJ software [8]. Cells were fixed with Karnovsky fixative, pelleted, and post-fixed with 1% osmium tetroxide. Cells were then serially dehydrated in ethanol, rinsed in propylene oxide, and incubated in a mixture of propylene oxide and resin prior to embedding. Samples were sliced, mounted on metal gratings, and imaged with the magnification of 50,000×. The shortest line between the outer mitochondrial membrane and ER was drawn to represent the minimum distance between the two organelles and measured using ImageJ as previously reported [8].

### 4.9. EdU Detection

EdU assay was used to assess cell proliferation and was performed according to the manufacturer’s instructions. Images were taken using an Olympus FV1000 confocal laser scanning microscope. Proliferating cells were detected and calculated as previously reported [36]. The number of EdU-positive cells was counted in random optical fields and the ratio of EdU-positive cells to Hoechst-stained cells was calculated.

### 4.10. Immunofluorescence Staining

The right lung tissues were embedded in OCT compound and snap-frozen with liquid nitrogen. The tissues were cut into 15-μm sections. Cells were washed twice with PBS and fixed with 4% PFA for 30 min at room temperature. The tissue sections were permeated with 0.1% Triton-X-PBS and subsequently blocked with normal serum for 1 h at room temperature. Diluted antibodies (NgBR and α-SMA) were added and maintained overnight at 4 °C. Cells and tissue sections were washed and incubated with secondary antibodies for 1 h at room temperature. Nuclei were stained with 4′,6-diamidino-2-phenylindole (DAPI; Beyotime). Confocal images were captured with an Olympus FV1000 microscope using AIM4.2 software. For analysis of NgBR expression in pulmonary arteries, NgBR fluorescence was excited at 543 nm and emission was 560–660 nm. VSMC marker α-SMA exciation was performed at 488 nm and emission was 505–525 nm. Then NgBR fluorescence was calculated using AIM4.2 software. For analysis of the MAM formation [12], cells were transfected with plasmids expressing mCherry fused with a targeting sequence of subunit IV of COX (from GeneChem). Protein disulfide isomerase (PDI; Abcam) was used as an ER marker. Pictures were acquired with the magnification of 1000×. Then the mCherry positive cells were used to calculate the Pearson’s correlation coefficient, which indicates the strength of mitochondria-ER interaction.

### 4.11. Rhod-2 AM Staining

Rhod-2 AM (Gibco, Invitrogen, Carlsbad, CA) staining was used to measure Ca^2+^_m_, and was performed according to the commercially available protocol. Cells were incubated with dihydrorhod-2 AM (4 μM) at 37 °C in the presence of 0.003% (w/v) pluronic acid for 40 min. After 0.5 h of de-esterification, cells were washed and incubated with mitochondrial green marker at 37 °C for 15 min. Then cells were washed and mounted. Rhod-2 fluorescence was excited at 543 nm and emission was 560–660 nm. MitoTracker Green exciation was performed at 488 nm and emission was 505–525 nm.

### 4.12. mROS Detection

MitoSOX™ Red mitochondrial superoxide indicator was used to quantify ROS production. Cells were treated with MitoSOX™ working solution (5 μM) and incubated for 15 min at 37 °C. Then, cells were gently washed three times with PBS, and incubated with mitochondrial green marker at 37 °C for 15 min. Images were taken using an Olympus FV1000 microscope. MitoSOX™ Red excitation was performed at 543 nm and emission was 560–660 nm. MitoTracker Green exciation was performed at 488 nm and emission was 505–525 nm. 

### 4.13. Mitochondrial Inner Membrane Potential

Mitochondrial inner membrane potential (ΔΨ) was measured by treating cells with 10 nM TMRM (Invitrogen) for 30 min at 37 °C. Then, cells were gently washed three times with PBS, and incubated with mitochondrial green marker at 37 °C for 15 min. Images were taken using an Olympus FV1000 microscipe. TMRM excitation was performed at 543 nm and emission was 560–660 nm. MitoTracker Green exciation was performed at 488 nm and emission was 505–525 nm. 

### 4.14. Mitochondrial Respiration

The oxygen consumption rate (OCR) was measured with an XF-96 extracellular flux analyzer (Seahorse Bioscience, Billerica, MA). Cells were seeded in 96-well plates at a density of 8,000 cells per well. After 24 h of transfection with shNgBR or NgBR OE plasmid, the plates were incubated at 37 °C in 5% CO_2_ for 48 h. OCR was measured under basal conditions and after the sequential addition of 25 µL oligomycin, 25 µL carbonyl cyanide 4-(trifluoromethoxy) phenylhydrazone (FCCP), and 25 µL antimycin A/rotenone to 175 µL medium. The final working concentrations reached to 2.4 µM (oligomycin), 0.3 µM (FCCP), and 0.6 µM (antimycin A/rotenone). The maximal respiration, basal respiration, and spare capacity were determined as previously described [37]. Briefly, basal OCR was calculated as OCR before oligomycin minus OCR after antimycin A/rotenone. Maximal OCR was calculated as the OCR after FCCP minus non-mitochondrial OCR. Spare capacity was calculated as the difference between maximal OCR after FCCP minus basal OCR. To allow comparison between different experiments, data were calculated as the fold of control group. Data are presented as the mean ± standard error of the mean (SEM).

### 4.15. Statistical Analysis

Individual experiments were performed at least three times. All data are presented as the mean ± SEM. The statistical analysis and graph were performed with SPSS 16.0 and GraphPad Prism6 software. Normality test was performed, and then statistical differences between groups were evaluated by one-way analysis of variance followed by Bonferroni’s test for multiple comparisons. For experiments comparing two groups containing one-way variance, a two-sided, unpaired Student’s *t*-test was performed to evaluate the differences. *p* < 0.05 was statistically significant. 

## Abbreviations

Ca^2+^_m_	mitochondrial Ca^2+^
ER	endoplasmic reticulum
FACL-4	Fatty-Acid-Coenzyme A Ligase, Long-Chain 4
GO	glucose oxidation
GRP75	glucose regulated protein 75
VSMC	vascular smooth muscle cell
HIF-1α	hypoxia-inducible factor 1-α
MAM	mitochondria-associated membranes
IP_3_R3	1,4,5-trisphosphate receptor type 3
mROS	mitochondria-derived reactive oxygen species
NgBR	Nogo-B receptor
PACS2	phosphofurin acidic cluster sorting protein 2
PCNA	proliferating cell nuclear antigen
PDI	Protein Disulfide Isomerase
VDAC1	voltage-dependent anion-selective channel 1
